# Functional pre-therapeutic evaluation by genome editing of variants of uncertain significance of essential tumor suppressor genes

**DOI:** 10.1186/s13073-021-00976-x

**Published:** 2021-11-09

**Authors:** Amandine Billaud, Louise-Marie Chevalier, Paule Augereau, Jean-Sebastien Frenel, Christophe Passot, Mario Campone, Alain Morel

**Affiliations:** 1 Université d’Angers, Inserm, CRCINA, SFR ICAT, F-49000 Angers, France; 2 Institut de Cancérologie de l’Ouest Nantes-Angers, F-49000 Angers, France; 3 Université de Nantes, Inserm, CRCINA, F-44000 Nantes, France

**Keywords:** Variants of uncertain significance, Genome editing, Functional testing, BRCA1, BRCA2, POLE, Cancer, Theranostic

## Abstract

**Background:**

Targeted therapies in oncology are promising but variants of uncertain significance (VUS) limit their use for clinical management and necessitate functional testing in vitro. Using *BRCA1* and *BRCA2* variants, which have consequences on PARP inhibitor sensitivity, and *POLE* variants, potential biomarkers of immunotherapy response, we developed a rapid functional assay based on CRISPR-Cas9 genome editing to determine the functional consequences of these variants having potentially direct implications on patients’ access to targeted therapies.

**Methods:**

We first evaluated the functional impact of 26 *BRCA1* and 7 *BRCA2* variants by editing and comparing NGS results between the variant of interest and a silent control variant. Ten of these variants had already been classified as benign or pathogenic and were used as controls. Finally, we extended this method to the characterization of *POLE* VUS.

**Results:**

For the 23 variants that were unclassified or for which conflicting interpretations had been reported, 15 were classified as functionally normal and 6 as functionally abnormal. Another two variants were found to have intermediate consequences, both with potential impacts on splicing. We then compared these scores to the patients’ responses to PARP inhibitors when possible. Finally, to prove the application of our method to the classification of variants from other tumor suppressor genes, we exemplified with three *POLE* VUS. Among them, two were classified with an intermediate functional impact and one was functionally abnormal. Eventually, four *POLE* variants previously classified in databases were also evaluated. However, we found evidence of a discordance with the classification, variant p.Leu424Val being found here functionally normal.

**Conclusions:**

Our new rapid functional assay can be used to characterize the functional implication of *BRCA1* and *BRCA2* variants, giving patients whose variants were evaluated as functionally abnormal access to PARP inhibitor treatment. Retrospective analysis of patients’ responses to PARP inhibitors, where accessible, was consistent with our functional score evaluation and confirmed the accuracy of our protocol. This method could potentially be extended to the classification of VUS from all essential tumor suppressor genes and can be performed within a timeframe compatible with clinical applications, thereby having a direct theranostic impact.

**Supplementary Information:**

The online version contains supplementary material available at 10.1186/s13073-021-00976-x.

## Background

Over the last two decades, the therapeutic options available in oncology have evolved towards therapies targeted based on tumoral genetic information [1]. These new treatments improve patient outcomes and have fewer adverse effects. However, the functional significance of many variants of targetable genes remains unknown. In the ClinVar [2] database, 237 934 variants of uncertain significance (VUS) are registered for the total set of genes considered (32 951 genes represented). Most are missense variants and potential splice site variants. With recent technical improvements and the development of whole-exome and whole-genome sequencing, this number of VUS is likely to rise still further in the coming years [3], with one in three variants classified as VUS overall, and 80% located on tumor suppressor genes [4]. As VUS affect treatment options and patient management, this trend highlights the need for a method of functional testing.

Variants of the *BRCA1* and *BRCA2* genes can be used to illustrate this crucial problem. These two tumor suppressor genes have many roles, mostly in genome protection via the homologous recombination (HR) pathway [5–7]. Inheritable mutations of *BRCA1/2* increase the risk of breast cancer (50–80%) and ovarian cancer (40–60%) [8, 9], and have also been implicated in prostate and pancreatic cancers. With the development of PARP inhibitors, pathogenic variants of these genes are now biomarkers of response to these treatments [10–12]. Their multiple interaction domains and protein partners account for the tremendous diversity of variants found in tumors. More than 2660 *BRCA1* and 4840 *BRCA2* VUS are registered in ClinVar, which means that respectively 35.7% and 45.7% of the total currently known *BRCA1* and *BRCA2* variants are VUS. However, databases, such as ClinVar (https://www.ncbi.nlm.nih.gov/clinvar/), BRCA exchange (https://brcaexchange.org/), and UMD [13] (Universal Mutation Database, http://www.umd.be/BRCA1/), mostly contain germline variants. Somatic variants, which may be detected in only one or two individuals, also exist and many such variants remain unreported. Functional testing in vitro is currently based on transcriptional activation [14, 15], HR activity [16, 17], and splicing [18–20]. However, such tests are not necessarily compatible with clinical management in terms of the time taken to obtain results to guide treatment.

Thus, CRISPR-Cas9 genome editing is a promising tool for meeting the challenge of VUS classification [21–23]. We first tested genome editing on diploid cells, but the efficiency of editing was low and clonal expansion, which is time-consuming, was required. Moreover, most of the clones were heterozygous, with highly variable sensitivity to PARP inhibitors depending on proliferation rates. We therefore used haploid HAP1 cells. Here, we adapted the concept developed in *BRCA1* variant characterization by saturation genome editing [24, 25] to the study of specific variants detected in our clinical testing laboratory. By comparing editing frequencies, by next-generation sequencing (NGS), between a variant of interest (testing variant) and a silent variant classified as benign, and therefore functionally normal (control variant), we were able to calculate a functional score and to evaluate the functional consequences of 23 *BRCA1/2* VUS and 10 *BRCA1/2* variants already classified as benign or pathogenic. Among those, 23 were characterized in our laboratory during somatic mutation testing in patients. In this retrospective analysis, where patients had received PARP inhibitors, their responses were compared to the functional score calculated for their variant. We finally extended our method to the evaluation of seven variants of *POLE*, another tumor suppressor gene biomarker for immunotherapy administration, demonstrating the potential utility of this approach for characterization of variants of others essential tumor suppressor genes. The essentiality of the *BRCA1*, *BRCA2,* and *POLE* genes in our haploid model [25, 26] was a key feature, making it possible to evaluate function rapidly, within less than 3 weeks, compatible with a direct benefit of the patient carrying the variant.

## Methods

### Population

Tumor suppressor gene (*BRCA1*, *BRCA2,* and *POLE*) somatic variants included in this study were selected solely based on their status of variants of uncertain significance or unclassified variants; well-known variants were also analyzed as controls. All of them were characterized by next-generation sequencing at the *Institut de Cancérologie de l’Ouest* (ICO, Angers, France). Consequently, 25 patients suffering from high-grade serous ovarian cancer were enrolled in this study, originating from the Institut de Cancerologie de l’Ouest and the Centre Hospitalier du Mans (Le Mans, France). The functional consequences of these selected variants were evaluated by genome editing in haploid cells and the results were compared to the patients’ response to PARP inhibitors where available. These patients were treated with PARP inhibitors at the ICO, allowing access to their treatment results. This retrospective study was approved by the local ethics committee of Angers medical university (France) under the reference 2020-05.

### HAP1 cell culture

Wild-type haploid HAP1 cells were purchased from Horizon Discovery and cultured in Isocove’s modified Dulbecco’s media (IMDM) containing L-glutamine and 25 mM HEPES (Corning), supplemented with 10% fetal calf serum (Eurobio). Cells were grown at 37 °C, under an atmosphere containing 5% CO_2_ and were passaged before confluence, to prevent reversion to the diploid state. Haploidy of HAP1 cells was confirmed by measure of DNA content via coloration with propidium iodide (PI) dye following Vindelov method [27] and cytometry analysis before their use.

### Genetically engineered HAP1 cells

Polyclonal LIG4 knock-out cells were generated with CRISPR-Cas9 technology [28]. Briefly, a guide RNA (gRNA) was first designed to target the second exon with an *AflIII* restriction site three nucleotides upstream from the PAM sequence. An Alt-R CRISPR-Cas9 crRNA (IDT DNA) (5′-CAATTACACAGTACGTGTCT-3′) and an Alt-R CRISPR-Cas9 tracrRNA with an ATTO550 fluorescent dye (IDT DNA) were complexed at a final concentration of 1 μM with 6 pmol of Alt-R S.p. Hifi Cas9 Nuclease V3 (IDT DNA) in presence of Lipofectamine CRISPRMAX Cas9 Transfection Reagent (Thermo Fisher Scientific). The mixture was incubated for 20 min, and reverse transfection was then performed by adding RNA-Cas9 ribonucleoprotein complexes to 1.6 × 10^5^ cells. Four hours after transfection, cells were sorted by FACS based on ATTO550 fluorescence. Only 20% of cells with the highest level of fluorescence were retained and used to seed with IMDM supplemented with 1% penicillin-streptomycin (Gibco). The cells were incubated for 5 days and then subjected to limiting dilution. About 20 clones were amplified for DNA extraction with Chelex 100 Resin (Biorad). We used 10 μL of these DNA extract for PCR amplification (forward primer: 5′- CTGGAGAACAGAATTGCAGA-3′; reverse primer: 5′-TAGCAATCATATTCACGGGC-3′) followed by digestion with the *AflIII* restriction enzyme (New England Biolabs) for 1 h at 37 °C. The mixture was then incubated for 20 min at 80 °C for enzyme inactivation. The clones were screened by following their migration in a 2% agarose gel on electrophoresis. Clones that had undergone genomic editing and had lost the restriction site were identified by Sanger sequencing on an ABI 3130 Genetic Analyzer (Thermo Fisher Scientific) with the BigDye Terminator v1.1 Cycle Sequencing Kit (Thermo Fisher Scientific). Results were visualized and analyzed with Sequencing Analysis 5.3.1 software (Thermo Fisher Scientific). The exact same protocol was followed to generate the polyclonal XRCC4 KO HAP1 cell line (RNAg and primer sequences are listed in Additional file 1: Table S1).

### VUS selection and gRNA design

First, ten variants of *BRCA1*, three of *BRCA2,* and two of *POLE* were selected for study based on their status as variants of uncertain significance or unclassified variants; 10 well-known variants of *BRCA1* (p.Tyr179Cys, p.Cys197=, p.Tyr422X, p.Gln1604X, p.Gln1604=, and p.Pro1812Ala) and *BRCA2* (p.Asp935Asn, p.Ser1882X, p.Val2728Ile and p.Gln2829Arg) were also analyzed as controls. All these 25 variants were characterized by NGS in our laboratory during somatic mutation testing in patients. To increase the number of characterized VUS, we also selected 10 variants of *BRCA1* (p.Ile31Asn, p.Glu149Ala, p.Val191Asp, p.Gln210=, p.Gly462Arg, p.Arg979Cys, p.Gly1201Ser, p.Thr1394Ile, p.Ala1752Pro, p.Gly1770Val) and a *POLE* variant (p.Arg1826Trp) for which conflicting interpretations had been reported in the databases (ClinVar [2], OncoKB [29]). Four variants of *POLE* (p.Ala31Ser, p.Pro286Ser, p.Leu424Val, p.Phe695Ile) were also selected in databases and used as controls (ClinVar, OncoKB). Alt-R CRISPR-Cas9 crRNA (IDT DNA) were designed with the Alt-R Custom Cas9 crRNA design tool (IDT DNA) (https://eu.idtdna.com/site/order/designtool/index/CRISPR_CUSTOM). The PAM sequence had to be adjacent to the variant to facilitate the editing of KO LIG4 HAP1 cells [30]. We also selected the gRNA based on the possibility of inserting a silent variant into the PAM sequence or the 3 to 5 nucleotides immediately upstream [30]. This increases editing efficiency and will be used as a control in subsequent experiments (reference variant). For each variant, we designed two Ultramer DNA Oligos (IDT DNA) of about 84 nt. The first contained the patient’s variant (testing variant) and the second contained a silent variant (control variant), already reported to be benign if possible, and therefore functionally normal. Both contained the silent reference variant mentioned above. All the gRNA and DNA oligonucleotides designed are reported in Additional file 1: Table S1.

### Transfection of LIG4 KO HAP1 cells

For each variant, two transfections were performed simultaneously, both with the same gRNA but with different DNA oligomers (the VUS to be classified in one transfection or the control variant in the other). We used the protocol described earlier for knocking-out *LIG4* in HAP1 cells, but with 2 nmol of DNA oligonucleotides added before the Lipofectamine CRISPRMAX Cas9 Transfection Reagent. A cell suspension containing 400,000 cells/mL in IMDM supplemented with 10% FBS was then prepared, and Alt-R HDR Enhancer (IDT DNA) was added to a final concentration of 2 nM. Reverse transfection was then performed. On day 1 post-transfection, the medium was replaced with fresh IMDM supplemented with 10% FBS. On day 4 to 5, depending on the degree of confluence, the cells were released by trypsin treatment and used to seed 6-cm-diameter plates. Two days after plating, a second transfection was performed with the same protocol for both types of transfection, to enrich the cell preparation in edited cells. The cells were then incubated for a further 4 to 5 days before DNA extraction.

### DNA extraction and NGS sequencing

All gDNA were extracted from edited cells with the Maxwell 16 Blood DNA Purification Kit (Promega) and quantified using a Qubit (Thermo Fisher Scientific) and the Quantifluor dsDNA System Kit (Promega). We then used 20 ng of the extracted DNA to generate the NGS library. The libraries were prepared with the Oncomine BRCA Assay Manual Kit (Thermo Fisher Scientific) or a custom assay Ampliseq for *POLE* (Thermo Fisher Scientific), allowing amplification of the entire *BRCA1* and *BRCA2* or *POLE* coding regions and noncoding putative splice boundaries. Then, 20 ng of DNA per sample was needed and barcoded, the libraries were subjected to clonal amplification by PCR emulsion with an Ion Chef System (Thermo Fisher Scientific) according to the manufacturer’s instructions. The prepared libraries were then sequenced on an Ion Torrent S5 Sequencer with the Ion 520 and 530 Chef Kit (Thermo Fisher Scientific).

Variants of interest were visualized with Integrative Genomics Viewer (IGV) [31]. Human Genome Variation Society (HGVS)-approved guidelines (http://www.hgvs.org/mutnomen/) were used. The variants found by NGS were researched in the UMD-BRCA1 or UMD-BRCA2 databases [13], the ClinVar database, the BRCA exchange database, and the BRCA Mutation Database (http://www.arup.utah.edu/database/BRCA/Home/BRCA1_landing.php) to assess their classification and functional impact where available.

### Sequencing analysis and VUS functional score evaluation

Following NGS sequencing, insertions or deletions located around the expected cleavage site, in the eight nucleotides centered on the PAM sequence or the seven nucleotides centered on the VUS, were also counted. Indel frequencies were then calculated by dividing the total amount of indels by the total number of reads. For the evaluation of SNV coverage, the ratio of the total numbers of reads for the VUS evaluated and the control SNV was calculated. Finally, functional scores for all the variants studied were calculated by comparing the sequence frequencies of all the inserted variants (VUS of interest, silent control SNV, and silent reference SNV) and the results contained in the available databases (UMD database, ClinVar, BRCA exchange).

The following formula was used:

Functional score = ½ ∗ ((log2((*f*_mut_ ∗ *f*PAM_mut_)/(*f*_sil_ ∗ fPAM_sil_))) + (log2((*f*_mut_ ∗ *f*PAM_sil_)/(*f*_sil_ ∗ *f*PAM_mut_))))

With *f*_mut_ for variant frequency of the VUS of interest, *f*PAM_mut_ for PAM variant frequency (or silent reference SNV) corresponding to the cells edited with the testing variant, *f*_sil_ for variant frequency of the silent control SNV, and *f*PAM_sil_ for variant frequency of the silent reference SNV corresponding to the control condition. Read covers and indel frequencies must be similar in the control condition and the tested condition. All the variants measured for a given variant must be localized on a same read.

### Statistics

Linear regression analyses were performed to compare insertions and deletions frequencies between the control condition (silent control variant) and the studied variant (testing variant). Mann-Whitney tests were conducted to assess the potential statistical significance of the differences between the functional scores of the different classifications of variants (benign, intermediate and deleterious). Thresholds delineating the intermediate classification were calculated following the evaluation of the standard deviation of the functional scores among the benign and deleterious variants. All statistical analyses were performed with GraphPad Prism analysis software.

## Results

### A comparison of editing frequencies between *BRCA1/2* variants and silent control SNV can be used for functional classification

In oncology, the functional testing of variants of uncertain significance has become a major issue in the context of access to targeted therapies. We used the example of *BRCA1/2* variants to develop a method based on the CRISPR-Cas9 method. First, we promoted DNA repair by homologous recombination after the endonuclease action of Cas9 by inhibiting the non-homologous end-joining pathway (NHEJ) [32] by knock-out of the *LIG4* gene. The gRNA targeting this gene was selected according to its proximity to the *AflIII* restriction site, which is located at the Cas9 double-stranded cleavage site (Additional file 1: Fig S1A and S1B). The clones that had lost the restriction site were assumed to have undergone editing and were subjected to Sanger sequencing (Additional file 1: Fig S1C-E). All six of the clones sequenced had been edited, but only three also presented a frameshift (clones 5, 8, and 17). These three clones were pooled together to constitute the polyclonal LIG4 knock-out HAP1 cell line, to prevent side effects and putative off-target effects associated with clonal selection.

**Fig. 1:**
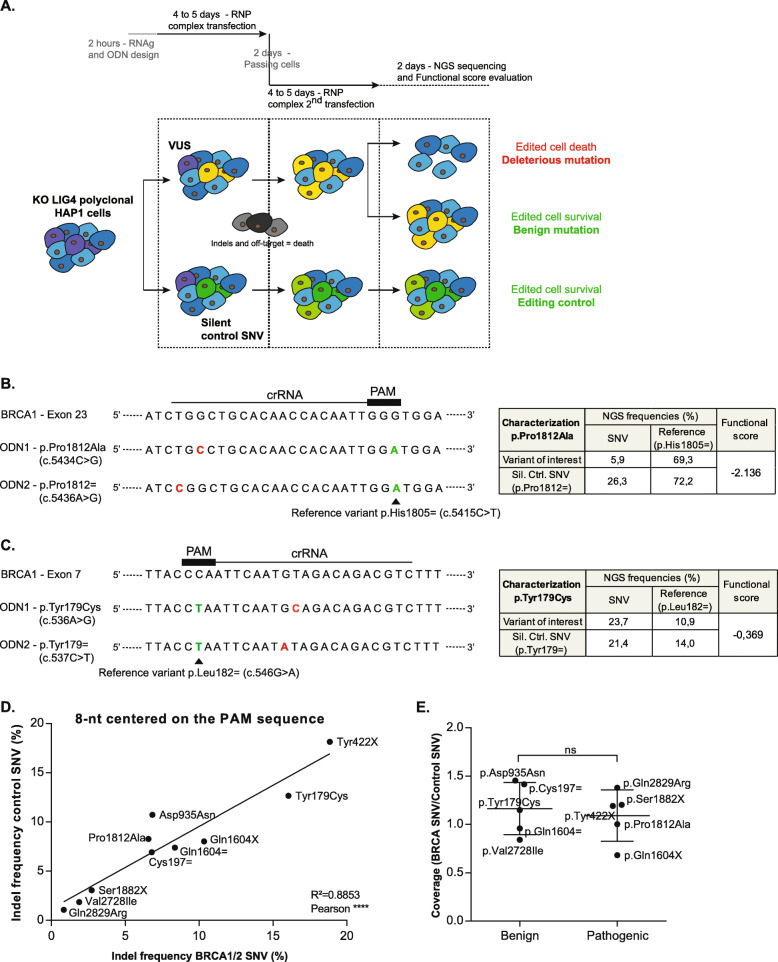
Comparison of editing frequencies between *BRCA1/2* variants and silent control SNVs for functional classification. **A** Experimental process used in this analysis for evaluation of the functional impact of *BRCA1/2* and *POLE* SNVs. **B** Examples of the gRNA and the two corresponding oligonucleotides designed for the p.Pro1812Ala variant editing, pathogenic mutation of the *BRCA1* gene. On this schematic representation, silent control and patient mutations of interest are colored in red, whereas silent reference variants are in green. NGS sequencing results and functional scores for this mutation are associated. **C** Same thing with the p.Tyr179Cys variant, benign variant of the *BRCA1* gene. **D** Insertion and deletion frequencies determined following the NGS sequencing of *BRCA1/2* variants of interest, compared with those for the corresponding silent control. These means include the 8 nt surrounding the PAM sequence. **E** Analysis of NGS sequencing coverage with the following formula: Mutation of interest (Mutation+Reference control)/Silent control (Mutation+Reference control). The results for benign *BRCA1/2* variants are compared with those obtained for pathogenic mutations (Mann-Whitney tests, *p* = 0.8016)

In HAP1 cells, *BRCA1* and *BRCA2* are essential genes [25, 26]. Genomic editing to create a functionally abnormal variant of these genes thus leads to cell death, facilitating the screening of edited cells. Moreover, edited cells with insertions or deletions instead of the variant of interest also die, due to the essential nature of the gene concerned. We checked that the absence of a variant following NGS sequencing was due to the functionality of the variant rather than a problem linked to genomic editing, by simultaneously performing a second transfection, with the same gRNA, but the insertion of a silent variant already classified as benign in databases, and therefore functionally normal, where possible (Fig. 1A). Thus, for each variant, we designed one gRNA and two oligonucleotides (Fig. 1B, C), one carrying the variant of interest, or testing variant, and the other carrying a silent control variant. We also added a second silent reference variant, also classified as benign where possible, located in the corresponding PAM motif or 3 nt upstream from the PAM motif. This second modification had two purposes. First, its insertion prevented from re-editing blocking gRNA association [30]. This second variant was present in both sets of conditions and was therefore also used as reference for comparisons. Following NGS sequencing, variant frequencies were compared and functional score was calculated (Fig. 1B, C). In our examples, the p.Pro1812Ala variant was classified as pathogenic in UMD and BRCA exchange databases and the p.Tyr179Cys variant as benign in UMD, ClinVar, and BRCA exchange databases. When the p.Tyr179Cys variant was analyzed, the frequency of the testing variants was similar to that of the control variant (Fig. 1C). On the contrary, for the p.Pro1812Ala variant, the frequency of the testing variant was considerably lower compared to the control variant (Fig. 1B) due to the essentiality of *BRCA1* gene.

**Fig. 2:**
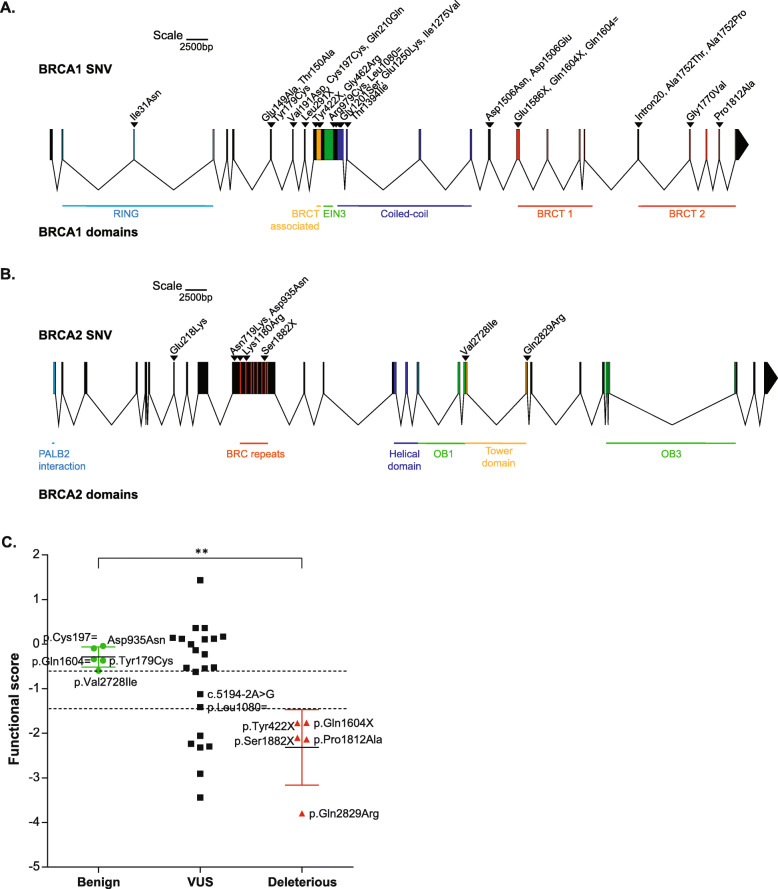
Functional characterization of *BRCA1/2* variants of unknown significance. **A** Schematic representation of the introns and exons of the *BRCA1* gene, showing the location of the variants selected for this analysis (http://wormw eb.org/exonintron). **B** Similar representation for the *BRCA2* gene. **C** Functional score evaluation after CRISPR-Cas9 editing and NGS sequencing of *BRCA1/2* variants of unknown significance and comparison with the functional scores obtained for functionally normal and abnormal mutations (Mann-Whitney tests, *p* = 0.0079)

We then validated our method testing 10 variants of *BRCA1* and *BRCA2* already classified as benign or pathogenic in databases. We evaluated the indel frequency to estimate the editing efficiency in the “testing variant” and “control variant” conditions compared for each variant. Indeed, this indel frequency was identical for both conditions when analyzed for the 8 nt surrounding the PAM sequence (Fig. 1D, *R*^2^ = 0.8853)) but not for the 7 nt surrounding the edited variant (patient or silent control variant) (Additional file 1: Fig S2, *R*^2^ = 0.0258). These results confirmed published findings; the Cas9 protein cleaves the DNA 3 nt upstream from the PAM sequence [33]. Moreover, the observed linear regression made it possible to evaluate genome editing efficiency and to compare the two conditions with the same gRNA. Following NGS sequencing, the coverages of the variant of interest (patient or silent control) and the reference control variant were also checked and shown to be similar in the two conditions (Fig. 1E). Finally, functional scores were calculated from sequencing frequencies (Fig. 2C and Table 1). The significant differences observed between benign and pathogenic variants validated the use of this method to value the function of variants of uncertain significance for the *BRCA1* and *BRCA2* genes. Functional score thresholds dividing variants into a functionally abnormal / intermediate / functionally normal classification were established from the analysis of these already classified variants by using the calculated standard deviations.

**Table 1:**
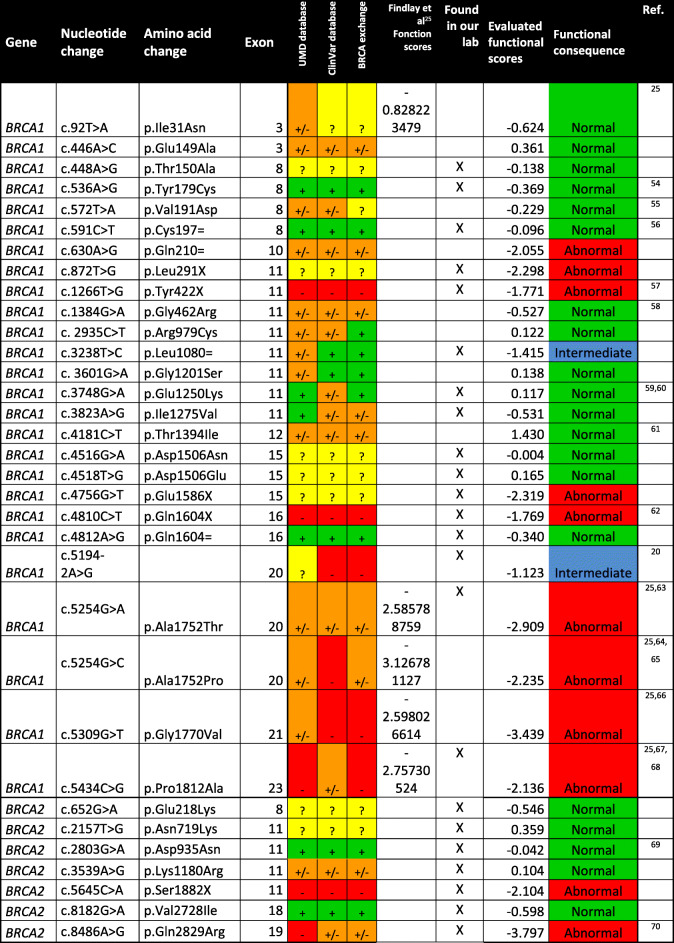
Functional scores for the evaluated *BRCA1/2* variants and comparison with database annotations

Variants classified as benign in databases (+) or functionally normal in our assay (Normal) are shown in green. Variants classified as pathogenic in databases (-) or functionally abnormal in our assay (Abnormal) are shown in red. Variants with conflicting interpretation are shown in orange (+/-), unreported mutations are shown in yellow (?). Variants with an intermediate score are shown in blue. Functional scores of variants already characterized by Findlay et al. [25] are included where available. Variants which were characterized in our laboratory are marked (X), the others were selected in the databases

### Assessment of validity of functional data according to guidelines

To assess the strength of evidence provided by our assay, we followed the four steps of the guidelines of Brnich et al. [34]. First, the disease mechanism seems reasonably well understood, since a loss of function of *BRCA1* and *BRCA2* genes is causal for homolog recombination deficiency [35]. Second, the used model seems appropriate since (i) the physiologic context seems reasonably respected in an haploid genome where the introduction of one variant is equivalent to the presence of the variant in a homozygous state in a diploid genome and (ii) the molecular consequence is very likely to be imputable to the variant itself since it is introduced by CRISPR-Cas9 genome editing and therefore no modification of regulatory sequences is expected. Third, the validity of the assay was evaluated with 10 controls (5 functionally normal and 5 functionally abnormal) and the OddsPath estimated with the table provided by Brnich et al. (Additional file 1: Table S2).

### Functional characterization of *BRCA1/2* variants of unknown significance

The fourth step of the guidelines, which is the application of evidence to individual variants, could then be performed. The strength of evidence was moderate for both PS3 (pathogenic) and BS3 (benign) categories with OddsPath > 4.3 and < 0.23 respectively. Thirteen variants of *BRCA1* and *BRCA2* were initially identified in our laboratory and considered as VUS based on an absence of annotation concerning their function. We then also studied other 10 variants of *BRCA1* previously classified as VUS or not previously reported in the databases (ClinVar, BRCA exchange, UMD) (Table 1). References mentioning these variants and their potential functional impact are listed in Table 1 where available [20, 25, 36–52]. These 23 VUS and the 10 other variants we used for validating our system affected different domains of the proteins and were distributed along the entire length of these genes (Fig. 2A, B). All reference silent variants, silent control variants, and their classifications in databases are listed in Additional file 1: Table S3. For each variant, we checked the coverage and indel frequencies (Additional file 1: Fig. S2B and S2C, *R*^2^ = 0.9328) for the testing variant and the control variant. All functional scores were then calculated from NGS frequencies (Fig. 2C and Table 1), and the variants were classified according to their impact on cell survival and, consequently, their functional impact in our model. Most of the 23 VUS analyzed were functionally normal (about 65.2%), but six were found to have an impact on edited cell survival (p.Gln210=, p.Leu291X, p.Glu1586X, p.Ala1752Thr, p.Ala1752Pro, p.Gly1770Val). Another two variants (p.Leu1080= and c.5194-2A>G) had intermediate functional classification.

### Patients’ responses to PARP inhibitor administration and comparison with the calculated functional scores

The next step was to confront our functional score to clinical data and the responses to PARP inhibitors of patients presenting alterations of *BRCA1/2*. The study we conducted was retrospective; hence, only 6 of the patients concerned received a PARP inhibitor treatment thus far. The mean age of these patients was 60.5 years and all of them suffered from high-grade serous ovarian cancer with different variants of *BRCA1* and *BRCA2*. The characteristics of their tumors are summarized in Table 2. Their responses to PARP inhibitors ranged from 3 months before resistance to 9 months and continuing. We then compared these data to the functional scores we had calculated for their variants. Five had, indeed, been classified as functionally abnormal and one with intermediate functional score (Tables 1 and 2). The lowest response to PARP inhibitor, from patient 4, was associated with this intermediate functional score.

**Table 2:** Baseline tumor characteristics of patients who were treated with PARP inhibitors

Samples	1	2	3	4	5	6
**Tumor histology**	High-grade serous ovarian carcinoma	High-grade serous ovarian carcinoma	High-grade serous ovarian carcinoma	High-grade serous ovarian carcinoma	High-grade serous ovarian carcinoma	High-grade serous ovarian carcinoma
**Tumor type**	Metastatic	Metastatic	Metastatic	Primary	Metastatic	Metastatic
**Tumor content (%)**	40	50	30	80	30	70
**Tumor fragment type**	Surgical sample	Biopsy	Biopsy	Surgical sample	Biopsy	Biopsy
**Gene**	BRCA1	BRCA1	BRCA1	BRCA1	BRCA1	BRCA2
**Exon**	11	15	16	20	23	11
**Allele frequency (%)**	8	23.5	19.9	75.7	75.9	61.7
**Nucleotide change**	c.872T>G	c.4756G>T	c.4810C>T	c.5194-2A>G	c.5434C>G	c.5645C>A
**AA change**	p.Leu291X	p.Glu1586X	p.Gln1604X	p.?	p.Pro1812Ala	p.Ser1882X
**Somatic or germline**	Somatic	Somatic	Somatic	Somatic	Germline	Germline
**Patients’ outcome following PARPi administration**	Maintenance treatment by PARPi (olaparb) since 7 months	Progression after 6 months on PARPi (niraparib)	Maintenance treatment by PARPi (olaparb) since 10 months	Progression after 3 months on PARPi (olaparib)	Maintenance treatment by PARPi (olaparb) since 7 months	Maintenance treatment by PARPi (olaparb) since 8 months
**Evaluated functional score**	− 2.298	− 2.319	− 1.769	− 1.123	− 2.136	− 2.104
**Functional consequence**	Functionally abnormal	Functionally abnormal	Functionally abnormal	Intermediate	Functionally abnormal	Functionally abnormal

### Extension of the experimental process to the functional evaluation of *POLE* variants

**Fig. 3:**
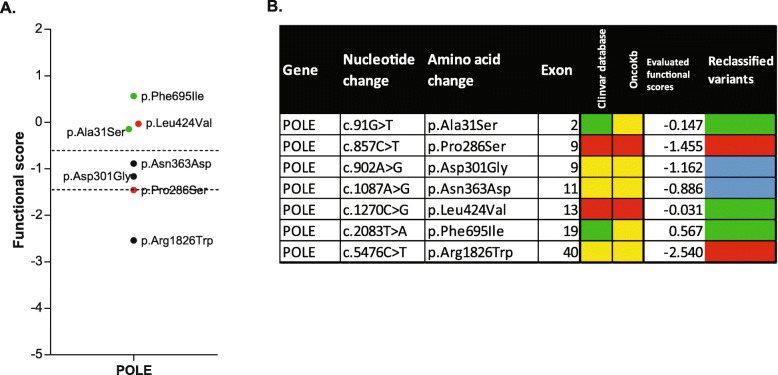
Extension of the experimental process to the functional evaluation of *POLE* variants. **A** Functional score evaluation following the CRISPR-Cas9 editing and NGS sequencing of *POLE* variants of unknown significance, and comparison with the scores obtained for two benign and two pathogenic mutations. **B** Table comparing the calculated functional scores of *POLE* variants and ClinVar and OncoKb databases classifications: mutations classified as benign in databases or functionally normal in our assay are shown in green, unreported mutations are shown in yellow, mutations classified as pathogenic in databases or functionally abnormal in our assay are shown in red and intermediate mutations are shown in blue

We then extended our protocol to the characterization of VUS from other tumor suppressor genes that were also essential in our model. We chose to study variants of the *POLE* gene because of potential interest of their functional impact for determining access to immunotherapy. We therefore selected seven *POLE* variants from databases, which included two classified as benign and two classified as pathogenic (Fig. 3B). The same protocol was followed to evaluate their functional scores, and the results obtained were compared with published findings (Fig. 3A, B). The p.Leu424Val variant, which has been classified as pathogenic in databases, was surprisingly found to have a functional score of − 0.031 rather associated with a functionally normal variant according to our experimental protocol and the cut-off we used.

## Discussion

Our evaluation of the functional scores of 33 variants of *BRCA1/2,* including 23 unreported or of uncertain significance, allows the classification of 20 of them as functionally normal, six as functionally abnormal (p.Gln210=, p.Leu291X, p.Glu1586X, p.Ala1752Thr, p.Ala1752Pro, p.Gly1770Val) and two as intermediate (p.Leu1080= and c.5194-2A>G) (Table 1). These results were consistent with databases (ClinVar, BRCA exchange, UMD), with previous functional studies (Table 1), where available, and with the published saturation genome editing study of the RING and BRCT domains of *BRCA1* (p.Ile31Asn, p.Ala1752Thr, p.Ala1752Pro, p.Gly1770Val and p.Pro1812Ala) [25]. Indeed, we evaluated, as expected following their publication, variant p.Ile31Asn as functionally normal whereas the four others were functionally abnormal.

Four of the six variants we classified as functionally abnormal were located in the BRCT domain of *BRCA1* (Fig. 2A), responsible of its interaction with various phosphorylated proteins such as Abraxas, BRIP1, or CtIP [6, 53]; the other two were previously unreported nonsense variants. The results were more surprising for the p.Gln210 = variant of *BRCA1*, which was also found functionally abnormal. However, the p.Gln210 = was described in databases as a variant possibly creating or strengthen a splice site of *BRCA1*, which is compatible with our results suggesting it is pathogenic. One of the two intermediate variants, c.5194-2A>G, has already been reported to affect splicing and may also be functionally abnormal. Its classification as functionally intermediate might reflect the existence of a large number of *BRCA1* splicing variants with a not totally deficient splicing site also called leaky splicing effect [54–56]. The sensitivity of the patient 4, who relapsed after only 3 months on PARP inhibitor, was consistent with these hypotheses (Fig. 3) and might also reflect the low level of expression of the splice variant compared to the full-length transcript despite the high variant frequency in the sample (analysis of the patient’s RNA by RT-qPCR and previously published) [20]. The second intermediate variant, p.Leu1080=, is located in the middle of exon 11 of *BRCA1*. This synonymous variant has not been reported in databases having a likelihood of resulting in a splicing alteration according to bioinformatic analyses. However, our intermediate functional score is consistent with the finding of the ESEfinder tool (http://krainer01.cshl.edu/cgi-bin/tools/ESE3/esefinder.cgi?process = home) [57, 58], a bioinformatic tool used to identify exonic splicing enhancers, which predicted that this variant might create a SRp40 ESE site. We cannot rule out the possibility that the cut-off used to classify the variants might be too low, but this value could be refined further after the analysis of more *BRCA1/2* variants. Moreover, all variants were compared to silent variants classified as non-pathogenic or as assumed to be benign. Following NGS, all these variants were indeed found to be functionally normal (Additional file 1: Table S3).

A 3-week period to determine the functional impact of a variant is compatible with clinical management and is one of the main advantages of our protocol (Fig. 1A). No clone selection is required, thus avoiding potential off-target effects that could occur in one cell. This genome editing-based protocol can be used to evaluate exonic, intronic, and even splicing variants. Moreover, the comparison between the calculated functional scores and the response of patients carrying these variants to PARP inhibitors were concordant, included for the intermediate score (Fig. 3). The main difficulty is finding a Cas9 PAM sequence (NGG) close enough to the variant to edit, to ensure binding of the gRNA. However, increasing numbers of Cas proteins from different species are becoming available, increasing PAM diversity [59]. We have already tested our protocol with the Cpf1 endonuclease. Similar results were obtained for the variant studied, implying a stable functional score, but editing efficiency in our hands was lower with this method. The need to improve editing efficiency may cease to be relevant with the appearance of base editing [60, 61] and, now, prime editing [62], which increase efficiency without the need for a double-strand break limiting off-target insertion and deletion frequencies. Moreover, prime editing will make functional characterization possible not only for point variants, but also for insertions or deletions. We have also generalized in another polyclonal cell line, knock-out for the *XRCC4* gene, also implicated in the NHEJ pathway [63] (Additional file 1: Fig. S3B and S3C). The same functional scores were obtained for the variants analyzed in this line, including p.Gln210= variant of *BRCA1*. However, more already classified variants should be analyzed to evaluate the thresholds in this other polyclonal cell line.

In this era of personalized medicine based on molecular genomics results provided by clinical laboratories, there is a need for harmonization of functional assay validity. To assess the validity of our assay, we followed the guidelines proposed by Brnich et al [34]. It allowed us to determine that our assay provides a “moderate” strength of evidence of the functional consequence for both pathogenic and benign variants.

We then extended our analysis to the characterization of another essential tumor suppressor gene: *POLE*, which is considered important for determining access to immunotherapy and four variants were used as control: two classified as benign and two as pathogenic. However, one of the pathogenic variants (p.Leu424Val) had a functional score typical of a functionally normal variant. We also analyzed three *POLE* VUS (Fig. 3A, B). Prediction algorithms in databases were conflicted concerning the consequences of p.Arg1826Trp variant for protein function: SIFT (deleterious) and PolyPhen-2 (probably damaging). The functional score of this variant was consistent with it being a functionally abnormal variant. Moreover, two VUS were classified as intermediate (p.Asn363Asp and p.Asp301Gly), but the cut-off used here should be refined by the evaluation of more well-known variants. Both these intermediate variants are located in the exonuclease domain and may therefore affect the proofreading activity of the protein, leading to a hypermutation phenotype [64]. The amino acids Asp301 and Asn363 are highly conserved between species. Both are predicted to affect function, but no functional tests have been performed. Moreover, the replacement of Asn with Asp in position 210 greatly decreases the endonuclease activity of *POLE* [65] and p.Asn363Asp variant has not been reported, but p.Asn363Lys is classified as pathogenic. The p.Asp301Gly and p.Asn363Asp variants, which had intermediate functional scores, may, therefore, actually be functionally abnormal. Moreover, the patient carrying the p.Asn363Asp variant had received immunotherapy treatment. However, he relapsed after only 6 months of continuous treatment which is consistent with our findings.

The principal difficulty encountered during this analysis concerned p.Leu424Val *POLE* variant, observed in multiple individuals with either an attenuated polyposis phenotype or a history of colorectal cancer [66–68]. This variant classified as pathogenic is also located in the exonuclease domain. However, the functional score obtained here suggested that it was more a functionally normal variant (Fig. 3). Leucine and valine have similar properties, so this substitution is generally considered conservative. According to an article on hypermutation, the mutation of leucine in position 424 has consequences very different to those of other well-known pathogenic variants of POLE (p.Pro436Ser or p.Pro286Arg) [69]. A strong mutation phenotype was observed when this residue was replaced with a proline or an isoleucine residue, but not when it was replaced with a valine. The mutation load associated with the loss of *POLE* proofreading activity therefore depending on the type of amino acid change involved, potentially accounting for our functional score. Alternatively, there may have been too little time before DNA extraction in our model for missense variants to accumulate, due to the loss of exonuclease activity. The loss of some genes may therefore require more time, despite their essentiality for highly deleterious effects on cell survival.

## Conclusions

The method presented here was proved effective for the characterization of the functional impact of *BRCA1* and *BRCA2* VUS and concordant with clinical data where available. More importantly, it can be used to obtain the necessary biological evidence of VUS function required for the prescription of targeted treatment within less than 3 weeks, which is compatible with use in clinical application. The patient carrying the genomic abnormality therefore benefits from an analysis of his or her variant, with potential consequences for relatives. This is particularly important for extremely rare somatic variants, which is compared to orphan diseases. The extension of its application to the study of *POLE* variants is already underway, and this method could be extended to the characterization of all essential tumor suppressor genes in our model [26], not only within the field of oncology. At a time at which purely in silico approaches are being used to guide therapeutic decisions, a method evaluating the functional implications of VUS is essential. Genomic editing is, thus, a promising tool for personalizing medicine and providing access to targeted therapy.

## Supplementary Information

**Additional file 1: Figure S1.** Generation of polyclonal LIG4 knock-out HAP1 cells. A. Schematic diagram of the LIG4 DNA region targeted by the gRNA, and localization of the AflIII restriction site used to knock out this gene in HAP1 cells. B. Schematic diagram of the protocol used to generate the polyclonal LIG4 KO cell line. C. Electrophoresis results following digestion with the AflIII restriction enzyme for screening of the clones. D. Sanger sequence alignments of the clones selected on basis of AflIII restriction site loss relative to HAP1 parental cells. E. Sanger electrophoregrams of LIG4 KO clones with insertions or deletions causing frameshifts relative to the HAP1 parental cell line. **Figure S2.** Analysis of coverage and frequencies of insertions and deletions obtained following genome editing and NGS sequencing of BRCA1 and BRCA2 variants. A. Insertion and deletion frequencies determined following the NGS sequencing of BRCA1/2 variants of interest, compared with those for the corresponding silent control. These means include the 7 nt surrounding the mutations of interest. B. Analysis of NGS sequencing coverage with the following formula: Mutation of interest (Mutation+Reference control)/Silent control (Mutation+Reference control). Results for all BRCA1/2 variants characterized by comparison with the classified benign and pathogenic mutations (Mann-Whitney tests, p = 0.1934 and p = 0.2902). C. Frequencies of insertions and deletions according to the NGS sequencing of BRCA1/2 variants and corresponding silent editing controls and linear regression analysis. These means include the 8 nt surrounding the PAM sequence. Variants classified in databases are shown in colours: benign mutations in green and pathogenic mutations in red. **Figure S3.** Evaluation of the reproducibility of our functional assay. A. Replicates of functions scores from four different variants obtained by following the described protocol in the LIG4 KO HAP1 cells (n = 3). Among these, the variants p.Pro1812Ala and p.Tyr422X were already classified as pathogenic in databases, the other two were VUS or had a conflicting interpretation. B. Sanger electrophoregrams of XRCC4 KO clones with insertions or deletions causing frameshifts relative to the HAP1 parental cell line. Clones 5 and 17 were pooled to generate the polyclonal XRCC4 KO HAP1 cell line. C. Comparison of the functional scores evaluated after CRISPR-Cas9 editing and NGS sequencing of four BRCA1 variants in polyclonal LIG4 KO cells and polyclonal XRCC4 KO cells. **Table S1.** gRNA and oligonucleotides sequences designed to edit *BRCA1*, *BRCA2* and *POLE* variants in the study. **Table S2.** Odds of Pathogenicity (OddsPath) estimated by performance of classified variant controls. In this assay, five benign and five pathogenic variants of *BRCA1* and *BRCA2* were used as controls allowing the estimation of the evidence strength of our classification according to Brnich et al [34] guidelines. **Table S3.** Variants used as silent controls or references with their classification according to different databases. Benign mutations are shown in green (+), unreported mutations are shown in yellow (?), mutations with conflicting interpretation are shown in orange (+/-), pathogenic mutations are shown in red (-) and intermediate mutations are shown in blue (I).

## Data Availability

The data that support the findings of this study are available within the manuscript or publicly deposited. Sequence data have been deposited in the European Nucleotide Archive (ENA) at EMBL-EBI under accession number PRJEB47293 (https://www.ebi.ac.uk/ena/browser/view/PRJEB47293) [70].

## Abbreviations

VUS: Variants of uncertain significance
HR: Homologous recombination
NGS: Next-generation sequencing
IMDM: Isocove’s modified Dulbecco’s media
PI: Propidium iodide
gRNA: Guide RNA
PAM: Palindromic adjacent motif
KO: Knock-out
IGV: Integrative genomics viewer
HGVS: Human genome variation society
Indels: Insertions or deletions
SNV: Single-nucleotide variant
NHEJ: Non-homologous end-joining
HDR: Homologous-directed repair
RING: Really interested new gene
BRCT: BRCA1 C terminus
