# Supplementary material for: Functional pre-therapeutic evaluation by genome editing of variants of uncertain significance of essential tumor suppressor genes
Source: Genome Med. 2021 Nov 9;13:174. doi: 10.1186/s13073-021-00976-x (PMC8576946; doi:10.1186/s13073-021-00976-x)
Supplement: Supplementary file 1 — Additional file 1: Figure S1. Generation of polyclonal LIG4 knock-out HAP1 cells. A. Schematic diagram of the LIG4 DNA region targeted by the gRNA, and localization of the AflIII restriction site used to knock out this gene in HAP1 cells. B. Schematic diagram of the protocol used to generate the polyclonal LIG4 KO cell line. C. Electrophoresis results following digestion with the AflIII restriction enzyme for screening of the clones. D. Sanger sequence alignments of the clones selected on basis of AflIII restriction site loss relative to HAP1 parental cells. E. Sanger electrophoregrams of LIG4 KO clones with insertions or deletions causing frameshifts relative to the HAP1 parental cell line. Figure S2. Analysis of coverage and frequencies of insertions and deletions obtained following genome editing and NGS sequencing of BRCA1 and BRCA2 variants. A. Insertion and deletion frequencies determined following the NGS sequencing of BRCA1/2 variants of interest, compared with those for the corresponding silent control. These means include the 7 nt surrounding the mutations of interest. B. Analysis of NGS sequencing coverage with the following formula: Mutation of interest (Mutation+Reference control)/Silent control (Mutation+Reference control). Results for all BRCA1/2 variants characterized by comparison with the classified benign and pathogenic mutations (Mann-Whitney tests, p = 0.1934 and p = 0.2902). C. Frequencies of insertions and deletions according to the NGS sequencing of BRCA1/2 variants and corresponding silent editing controls and linear regression analysis. These means include the 8 nt surrounding the PAM sequence. Variants classified in databases are shown in colours: benign mutations in green and pathogenic mutations in red. Figure S3. Evaluation of the reproducibility of our functional assay. A. Replicates of functions scores from four different variants obtained by following the described protocol in the LIG4 KO HAP1 cells (n = 3). Among these, the variants [file 13073_2021_976_MOESM1_ESM.pdf]

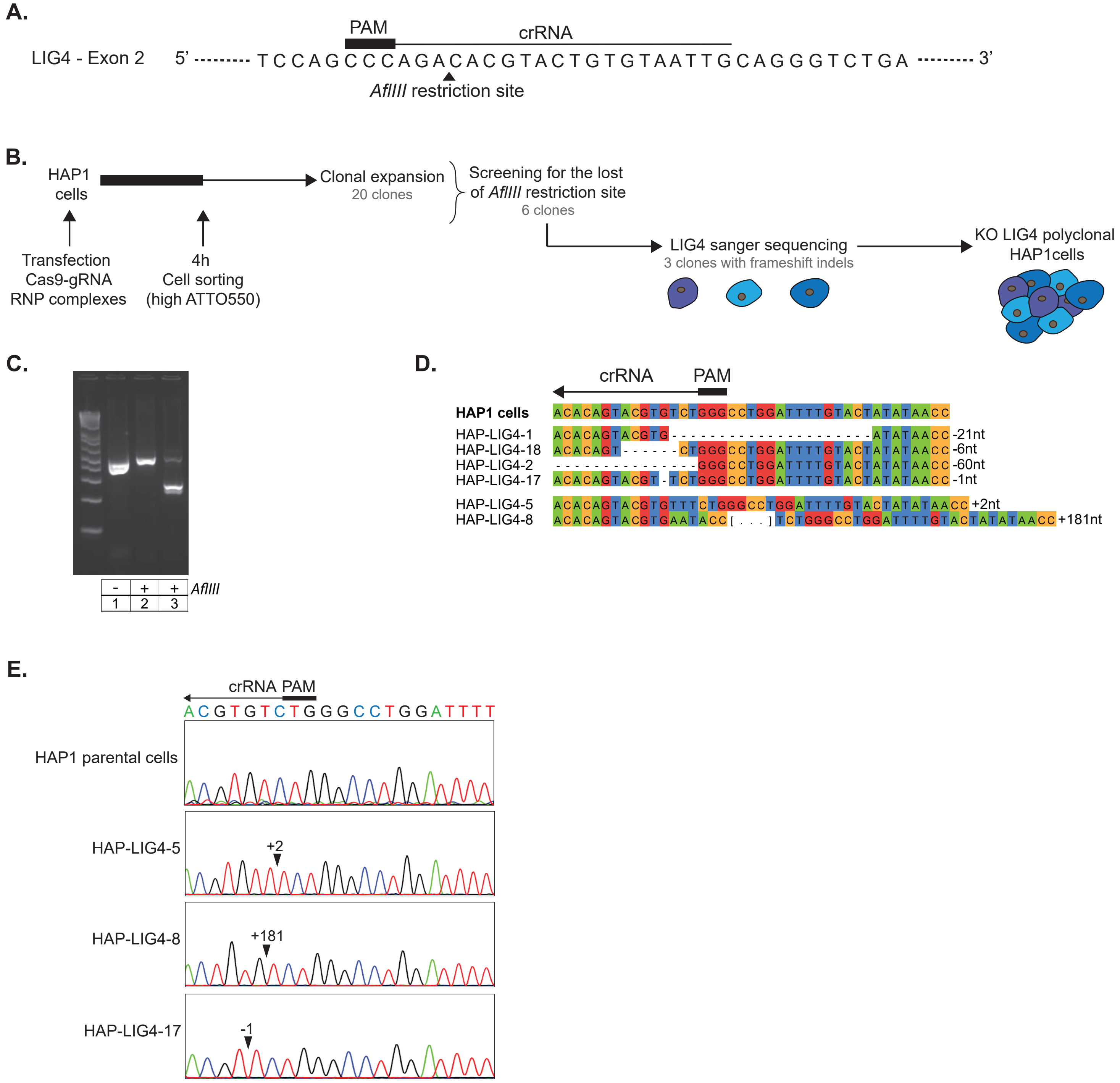

Figure S1: Generation of polyclonal LIG4 knock-out HAP1 cells. A. Schematic diagram of the LIG4 DNA region targeted by the gRNA, and localization of the AflIII restriction site used to knock out this gene in HAP1 cells. **B.** Schematic diagram of the protocol used to generate the polyclonal LIG4 KO cell line. **C.** Electrophoresis results following digestion with the AflIII restriction enzyme for screening of the clones. **D.** Sanger sequence alignments of the clones selected on basis of AflIII restriction site loss relative to HAP1 parental cells. **E.** Sanger electrophoregrams of LIG4 KO clones with insertions or deletions causing frameshifts relative to the HAP1 parental cell line.

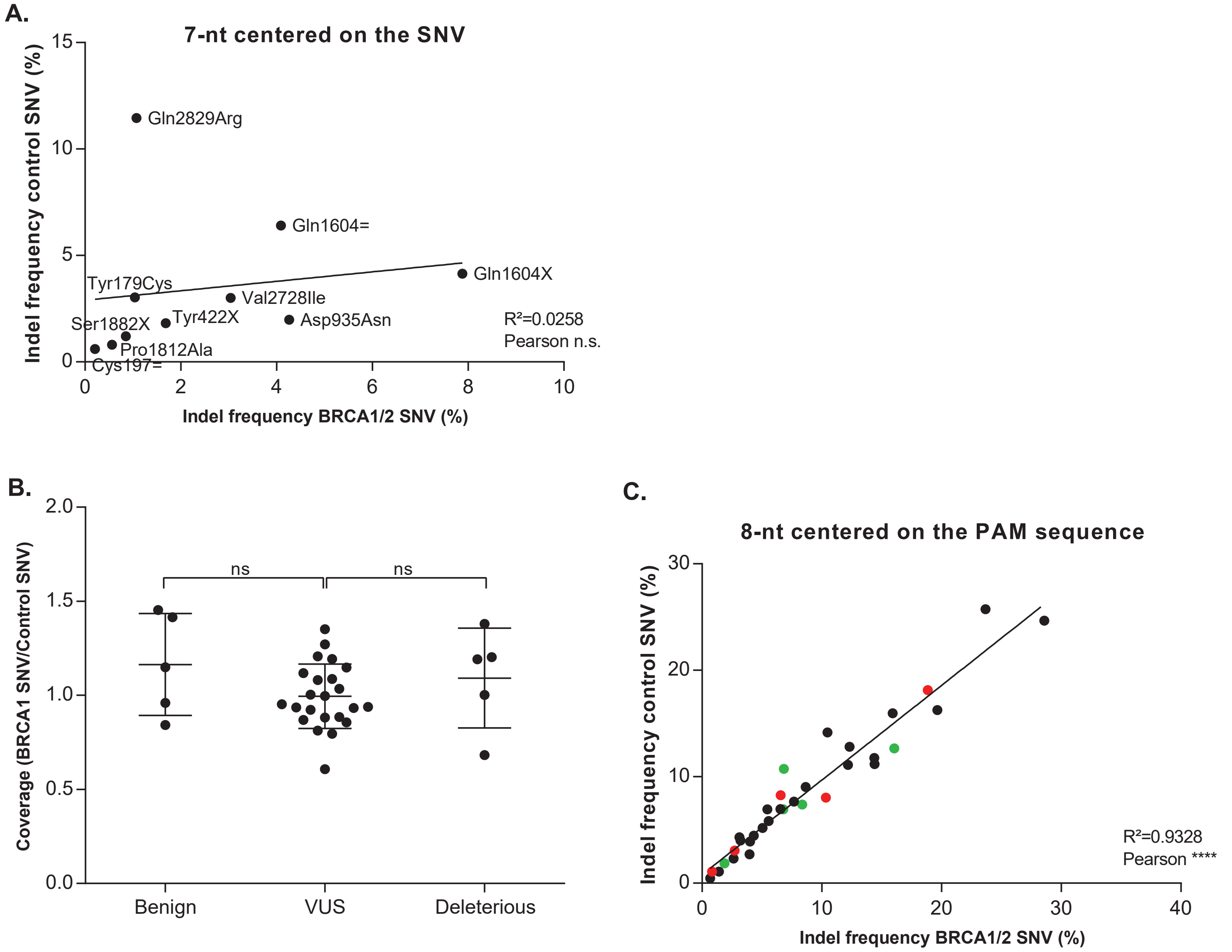

Figure S2: Analysis of coverage and frequencies of insertions and deletions obtained following genome editing and NGS sequencing of *BRCA1* and *BRCA2* variants. A. Insertion and deletion frequencies determined following the NGS sequencing of BRCA1/2 variants of interest, compared with those for the corresponding silent control. These means include the 7 nt surrounding the mutations of interest. **B.** Analysis of NGS sequencing coverage with the following formula: Mutation of interest (Mutation+Reference control)/Silent control (Mutation+Reference control). Results for all BRCA1/2 variants characterized by comparison with the classified benign and pathogenic mutations (Mann-Whitney tests, p=0.1934 and p=0.2902). **C.** Frequencies of insertions and deletions according to the NGS sequencing of BRCA1/2 variants and corresponding silent editing controls and linear regression analysis. These means include the 8 nt surrounding the PAM sequence. Variants classified in databases are shown in colours: benign mutations in green and pathogenic mutations in

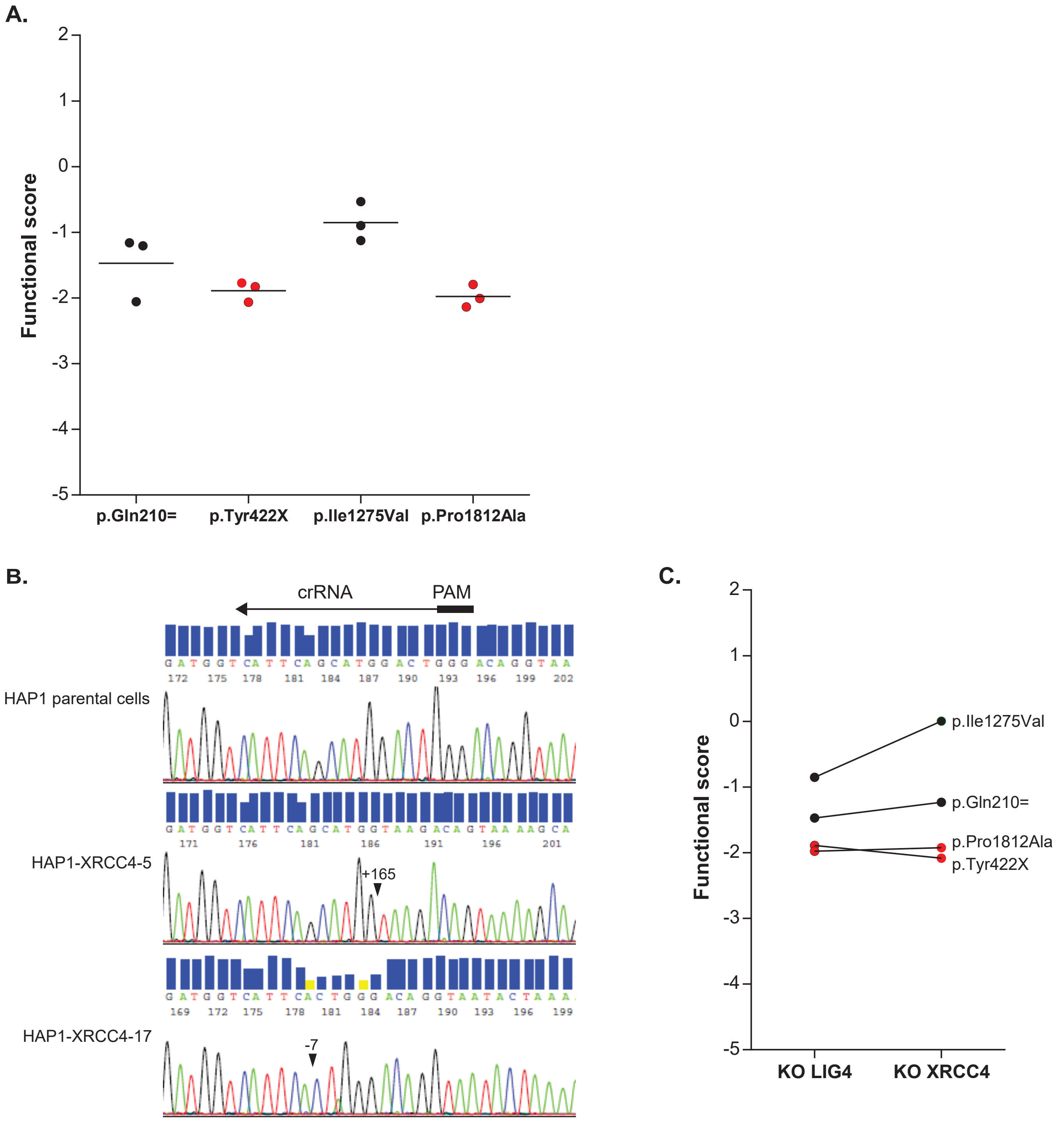

Figure S3: Evaluation of the reproducibility of our functional assay. A. Replicates of functions scores from four different variants obtained by following the described protocol in the LIG4 KO HAP1 cells (n=3). Among these, the variants p.Pro1812Ala and p.Tyr422X were already classified as pathogenic in databases, the other two were VUS or had a conflicting interpretation. **B.** Sanger electrophoregrams of XRCC4 KO clones with insertions or deletions causing frame-shifts relative to the HAP1 parental cell line. Clones 5 and 17 were pooled to generate the polyclonal XRCC4 KO HAP1 cell line. **C.** Comparison of the functional scores evaluated after CRISPR-Cas9 editing and NGS sequencing of four BRCA1 variants in polyclonal LIG4 KO cells and polyclonal XRCC4 KO cells.

Table S1: crRNA and oligonucleotides sequences designed to edit *BRCA1*, *BRCA2* and *POLE* variants in the study.

Genes	Names	crRNA sequences
BRCA1	BRCA1-Ile31Asn	TGCTAGTCTGGAGTTGATCA
BRCA1	BRCA1-Glu149Ala	TGGCTTCCTGCTAAACAGTA
BRCA1	BRCA1-Thr150Ala	TGGCTTCCTGCTAAACAGTA
BRCA1	BRCA1-Tyr179Cys	GACGTCTGTCTACATTGAAT
BRCA1	BRCA1-Val191Asp	TTCTGAAGATACCGTTAATA
BRCA1	BRCA1-Cys197=	TTCTGAAGATACCGTTAATA
BRCA1	BRCA1-Gln210=	ATCCAAACTGATTTCATCCC
BRCA1	BRCA1-Leu291X	GTTCTCATGCTGTAATGAGC
BRCA1	BRCA1-Tyr422X	TGTATTGGACGTTCTAAATG
BRCA1	BRCA1-Gly462Arg	TGGGAAAACCTATCGGAAGA
BRCA1	BRCA1-Arg979Cys	AAAGTGGTGGTATACGATAT
BRCA1	BRCA1-Leu1080=	GAATGCTATGCTTAGATTAG
BRCA1	BRCA1-Gly1201Ser	CCCTTTCACCCATACACATT
BRCA1	BRCA1-Glu1250Lys	AGACAGACACTCGGTAGCAA
BRCA1	BRCA1-Ile1275Val	TAACCAGGTAATATTGGCAA
BRCA1	BRCA1-Thr1394Ile	ACACACGCTTTTTACCTGAG
BRCA1	BRCA1-Asp1506Asn	ACCACCTATCATCTAATGAT
BRCA1	BRCA1-Asp1506Glu	ACCACCTATCATCTAATGAT
BRCA1	BRCA1-Glu1586X	GCCAACACGAGCTGACTCTG
BRCA1	BRCA1-Gln1604X	TTGGGGAACTTTCAATGCAG
BRCA1	BRCA1-Gln1604=	TTGGGGAACTTTCAATGCAG
BRCA1	BRCA1-Intron20	GAAACCAAACACAACCCATC
BRCA1	BRCA1-Ala1752Thr	AAAGCGAGCAAGAGAATCCC
BRCA1	BRCA1-Ala1752Pro	AAAGCGAGCAAGAGAATCCC
BRCA1	BRCA1-Gly1770Val	ACCTGTGGGCATGTTGGTGA
BRCA1	BRCA1-Pro1812Ala	CTGGCTGCACAACCACAATT
BRCA2	BRCA2-Glu218Lys	CTTACAGCAGTAGTATCATG
BRCA2	BRCA2-Asn719Lys	TGATTCTCTGTCATGCCTGC
BRCA2	BRCA2-Asp935Asn	ATGGTTTTATATGGAGACAC
BRCA2	BRCA2-Lys1180Arg	TGTCTACCTGACCAATCGAT
BRCA2	BRCA2-Ser1882X	AACCTGCCATAATTTTCGTT
BRCA2	BRCA2-Val2728Ile	AGATGGGTGGTATGCTGTTA
BRCA2	BRCA2-Gln2829Arg	TCAAAGAGCATACCCTATAC
POLE	POLE-Ala31Ser	AGTTTCGGCACTCAAGCGCC
POLE	POLE-Pro286Ser	TGTAGGAAATCATCATAATC
POLE	POLE-Asp301Gly	CACCTGGCCATCGATCATGT
POLE	POLE-Asn363Asp	CATCATGGTCACCTACAACG
POLE	POLE-Leu424Val	GGGCAGTCATAATCTCAAGG
POLE	POLE-Ala456Pro	GAATACGTGGCCAGAGTCTG
POLE	POLE-Phe695Ile	CCATCGGATCCAGCACCAGC

POLE	POLE-Arg1826Trp	AGCGGTAGAAGTGCATCACC
LIG4	LIG4-KO	CAATTACACAGTACGTGTCT
XRCC4	XRCC4-KO	ATGGTCATTCAGCATGGACT
**Genes**	**Names**	**Donor sequences**
BRCA1	BRCA1-Ile31Asn-mut	GCAAAATATGTGGTCACACTTTGTGGAGACAGGTTCTTTGTTCAACTCCAGACTAGCAGGGTAGGGGGGGAGAAAAAGAAAATAAATGAGGC
BRCA1	BRCA1-Ile31Asn-sil	GCAAAATATGTGGTCACACTTTGTGGAGACAGGTTCTTTAATCAACTCCAGACTAGCAGGGTAGGGGGGGAGAAAAAGAAAATAAATGAGGC
BRCA1	BRCA1-Glu149Ala-mut	CCAAGGTTAGAGAGTTGGACACTGAGACTGGTTGCCTGCTAAACAGTATGATAAAGAACAGTCAAGCAATTGTTGGCCAGTTCTGTGC
BRCA1	BRCA1-Glu149Ala-sil	CCAAGGTTAGAGAGTTGGACACTGAGACTCGTTTCCTGCTAAACAGTATGATAAAGAACAGTCAAGCAATTGTTGGCCAGTTCTGTGC
BRCA1	BRCA1-Thr150Ala-mut	CCAAGGTTAGAGAGTTGGACACTGAGACTGGCTTCCTGCTAAACAGTATGATAAAGAACAGTCAAGCAATTGTTGGCCAGTTCTGTGC
BRCA1	BRCA1-Thr150Ala-sil	CCAAGGTTAGAGAGTTGGACACTGAGACTCGTTTCCTGCTAAACAGTATGATAAAGAACAGTCAAGCAATTGTTGGCCAGTTCTGTGC
BRCA1	BRCA1-Tyr179Cys-mut	GCAATTATTATTAAATACTTAAAAAACCTGAGACCCTTACCTAATTCAATGCAGACAGACGTCTTTTGAGGTTGTATCCGCTGC
BRCA1	BRCA1-Tyr179Cys-sil	GCAATTATTATTAAATACTTAAAAAACCTGAGACCCTTACCTAATTCAATATAGACAGACGTCTTTTGAGGTTGTATCCGCTGC
BRCA1	BRCA1-Val191Asp-mut	GGTTCTCTTTGACTCACCTGCAATAAGTTGCTTTATTATCGGTATCTTCAGAAGAATCAGATCCTAAAAAATTTCCCCCC
BRCA1	BRCA1-Val191Asp-sil	GGTTCTCTTTGACTCACCTGCAATAAGTTGCTTTATTTACGGTATCTTCAGAAGAATCAGATCCTAAAAAATTTCCCCCC
BRCA1	BRCA1-Cys197=-mut	CCAGCTTCATAGACAAAGGTTCTCTTTGACTCACCTACAATAAGTTGCTTTATTAACGGTATCTTCAGAAGAATCAGATCC
BRCA1	BRCA1-Cys197=-sil	CCAGCTTCATAGACAAAGGTTCTCTTTGACTCACCTGCAGTAAGTTGCTTTATTAACGGTATCTTCAGAAGAATCAGATCC
BRCA1	BRCA1-Gln210=-mut	GCCATTACCCTTTTTTGCAGAATCCAAACTGATTTCATCCCTCGTTCCCTGAGGGGTGATTTGTAACAATTCTTGATCTCCC
BRCA1	BRCA1-Gln210=-sil	GCCATTACCCTTTTTTGCAGAATCCAAACTGATTTCATCCCTCGTACCTTGAGGGGTGATTTGTAACAATTCTTGATCTCCC
BRCA1	BRCA1-Leu291X-mut	CTACATTCATTCTGTCTTTAGTGAGTCATAAACTGCTGTTCTCATGCTGTAATGAGCTTGCATGAGTATTTGTGCCACATGGCTCC
BRCA1	BRCA1-Leu291X-sil	CTACATTCATTCTGTCTTTAGTGAGCAATAAACTGCTGTTCTCATGCTGTAATGAGCTTGCATGAGTATTTGTGCCACATGGCTCC
BRCA1	BRCA1-Tyr422X-mut	CACTGGCCAGTAAGTCTATTTTCTCTGAAGAACCAGACTATTCATCTACTTCATTTAGAACGTCCAATACATCAGCTACTTTGGC
BRCA1	BRCA1-Tyr422X-sil	CACTGGCCAGTAAGTCTATTTTCTCTGAAGAACCAGAGTATTCATCTACTTCATTTAGAACGTCCAATACATCAGCTACTTTGGC
BRCA1	BRCA1-Gly462Arg-mut	CAGTTACATGGCTTAAGTTGGGGAGGCTTGCTTTCTTCCGATAGGTTTTCCTAAATATTTTGTCTTCAATATTACTCTCTACTG
BRCA1	BRCA1-Gly462Arg-sil	CAGTTACATGGCTTAAGTTGGGGAGGCTTGCTTTCTTCCGATAGGTTTTTCCAAATATTTTGTCTTCAATATTACTCTCTACTG
BRCA1	BRCA1-Arg979Cys-mut	CTTACATTTAGTTTTAACAAATGACTTGATGGGAAAAAGTGGTGGTATACAATATGGATTTTGTAAAAGTCCATGTTTATTTGG
BRCA1	BRCA1-Arg979Cys-sil	CTTACATTTAGTTTTAACAAATGACTTGATGGGAAAAAGTGGTGGTATGCGATATGGATTTTGTAAAAGTCCATGTTTATTTGG
BRCA1	BRCA1-Leu1080=-mut	GGAAGACTTTGTTTATAGACCTCAGGTTGCAAAACTCCTAATCTAAGCATAGCATTCAGTTTTGGCCCTCTGTTTCTACCTAGTTCTGC
BRCA1	BRCA1-Leu1080=-sil	GGAAGACTTTGTTTATAGACCTCAGGTTGCAAAACACCTAATCTAAGCATAGCGTTCAATTTTGGCCCTCTGTTTCTACCTAGTTCTGC
BRCA1	BRCA1-Gly1201Ser-mut	GGACTCTAATTTCTTGGCCCCTCTTCGGTAACTCTGAGCGAAATGTGTATGGGTGAAAGGGCTAGGACTCCTGCTAAGCTCTCC
BRCA1	BRCA1-Gly1201Ser-sil	GGACTCTAATTTCTTGGCCCCTCTTCGGTATCCCTGAGCGAAATGTGTATGGGTGAAAGGGCTAGGACTCCTGCTAAGCTCTCC
BRCA1	BRCA1-Glu1250Lys-mut	GCTATTCTTCAATGATAATAAATTCTCCTCTGTGTTCTTAGACAGACACTTGGTAGCAACAGTGCTATGCCTAGTAGACTGAGAAGG
BRCA1	BRCA1-Glu1250Lys-sil	GCTATTCTTCAATGATAATAAATTCTCCTCTGTGTTCTTAGACAGACATTCGGTAGCAACAGTGCTATGCCTAGTAGACTGAGAAGG
BRCA1	BRCA1-Ile1275Val-mut	CCTCACTAAGGTGATGTTCCTGAGATGCTTTTGCCAATACTACCTGGTTACTGCAGTCATTTAAGCTATTCTTCAATGATAATAAATTCTCC
BRCA1	BRCA1-Ile1275Val-sil	CCTCACTAAGGTGATGTTCCTGAGATGCTTTTGCCAAGATTACCTGGTTACTGCAGTCATTTAAGCTATTCTTCAATGATAATAAATTCTCC
BRCA1	BRCA1-Thr1394Ile-mut	GCAAAGGACACCACACACACGCATGTGCACACACACACACGCTTTTTACCTGAATTGTTAAAATGTCACTCTGAGAGGATAGCCC

BRCA1	BRCA1-Thr1394Ile-sil	GCAAAGGACACCACACACACGCATGTGCACACACACACACGCTTTTTACCTGAGTTGTTAAGATGTCACTCTGAGAGGATAGCCC
BRCA1	BRCA1-Asp1506Asn-mut	CTCCCAGAGCAACTGTGCATGTACCACCTATTATCTAATGATGGACATTTAGAAGGGGATGACCTAGAAAGATAAATGGAAGG
BRCA1	BRCA1-Asp1506Asn-sil	CTCCCAGAGCAACTGTGCATGTACCACCTGTCATCTAATGATGGACATTTAGAAGGGGATGACCTAGAAAGATAAATGGAAGG
BRCA1	BRCA1-Asp1506Glu-mut	CTCCCAGAGCAACTGTGCATGTACCACCTCTCATCTAATGATGGACATTTAGAAGGGGATGACCTAGAAAGATAAATGGAAGG
BRCA1	BRCA1-Asp1506Glu-sil	CTCCCAGAGCAACTGTGCATGTACCACCTGTCATCTAATGATGGACATTTAGAAGGGGATGACCTAGAAAGATAAATGGAAGG
BRCA1	BRCA1-Glu1586X-mut	GCAGAGGTTGAAGATGGTATGTTGCCAACACGAGCTGACTATGGAGCTCTGTCTTCAGAAGGATCAGATTCAGGGTCATCAGAG
BRCA1	BRCA1-Glu1586X-sil	GCAGAGGTTGAAGATGGTATGTTGCCAACACGAGCTGATTCTGGAGCTCTGTCTTCAGAAGGATCAGATTCAGGGTCATCAGAG
BRCA1	BRCA1-Gln1604X-mut	GCAGCAGCTGGACTCTGGGCAGATTCTGCAACTTTCAATTAGGGAACTTTCAATGCAGACGTTGAAGATGGTATGTTGCCAACACGAGCTGACTCTGGGGC
BRCA1	BRCA1-Gln1604X-sil	GCAGCAGCTGGACTCTGGGCAGATTCTGCAACTTTCAACTGGGGAACTTTCAATGCAGACGTTGAAGATGGTATGTTGCCAACACGAGCTGACTCTGGGGC
BRCA1	BRCA1-Gln1604=-mut	GCAGCAGCTGGACTCTGGGCAGATTCTGCAACTTTCAACTGGGGAACTTTCAATGCAGACGTTGAAGATGGTATGTTGCCAACACGAGCTGACTCTGGGGC
BRCA1	BRCA1-Gln1604=-sil	GCAGCAGCTGGACTCTGGGCAGATTCTGCAACTTTCAATTGTGGAACTTTCAATGCAGACGTTGAAGATGGTATGTTGCCAACACGAGCTGACTCTGGGGC
BRCA1	BRCA1-Intron20-mut	CCATTGACCACATCTCCTCTGACTTCAAAATCATGCCGAAAGAAACCAAACACAACCCATCACGATAAGAGAAAGAGAAGCTTCCTTCAATGG
BRCA1	BRCA1-Intron20-sil	CCATTGACCACATCTCCTCTGACTTCAAAATCATGCTGAAAGAACCCAAACACAACCCATCACGATAAGAGAAAGAGAAGCTTCCTTCAATGG
BRCA1	BRCA1-Ala1752Thr-mut	GAGGGAGGGAGCTTTACCTTTCTGTCTTGGGATTCTCTTGGTCGCTTTGGACCTTGGTGGTTTCTTCCATTGACCACATCTCC
BRCA1	BRCA1-Ala1752Thr-sil	GAGGGAGGGAGCTTTACCTTTCTGTCTTGGGATTCTCTAGCTCGCTTTGGACCTTGGTGGTTTCTTCCATTGACCACATCTCC
BRCA1	BRCA1-Ala1752Pro-mut	GAGGGAGGGAGCTTTACCTTTCTGTCTTGGGATTCTCTTGTTCGCTTTGGACCTTGGTGGTTTCTTCCATTGACCACATCTCC
BRCA1	BRCA1-Ala1752Pro-sil	GAGGGAGGGAGCTTTACCTTTCTGTCTTGGGATTCTCTAGCTCGCTTTGGACCTTGGTGGTTTCTTCCATTGACCACATCTCC
BRCA1	BRCA1-Gly1770Val-mut	GGAACTCTGGGGTTCTCCCAGGCTCTTACCTGTGGGCATGTTGGTGAACGGCACATAGCAACAGATTTCTAGCCCCCTGAAGATCTGG
BRCA1	BRCA1-Gly1770Val-sil	GGAACTCTGGGGTTCTCCCAGGCTCTTACCTGTGGGCATGTTGGTGAACGGACCATAGCAACAGATTTCTAGCCCCCTGAAGATCTGG
BRCA1	BRCA1-Pro1812Ala-mut	GGAAGCCATTGTCCTCTGTCCAGGCATCTGCCTGCACAACCACAATTGGATGGACACCCTGGATCCCCAGGAAGGAAAGAGCATTC
BRCA1	BRCA1-Pro1812Ala-sil	GGAAGCCATTGTCCTCTGTCCAGGCATCCGGCTGCACAACCACAATTGGATGGACACCCTGGATCCCCAGGAAGGAAAGAGCATTC
BRCA2	BRCA2-Glu218Lys-mut	CAGTCAGAAATGAAGAAGCATCTAAAACTGTATTTCCTCACGATACTACTGCTGTAAGTAAATATGACATTGATTAGACTGTTG
BRCA2	BRCA2-Glu218Lys-sil	CAGTCAGAAATGAAGAAGCATCTGAGACTGTATTTCCTCACGATACTACTGCTGTAAGTAAATATGACATTGATTAGACTGTTG
BRCA2	BRCA2-Asn719Lys-mut	GTTATTTATTACCCCAGAAGCTGATTCTCTGTCATGCCTGCAAGAAGGACAGTGTGAAAAGGATCCAAAAAGCAAAAAAGTTTCAG
BRCA2	BRCA2-Asn719Lys-sil	GTTATTTATTACCCCAGAAGCTGATTCTCTGTCATGCCTGCAAGAAGGACAGTGTGAAAACGATCCAAAAAGCAAAAAAGTTTCAG
BRCA2	BRCA2-Asp935Asn-mut	CGAACCCATTTTCAAGAACTCTACCATGGTTTTATATGGAGATACAGGTAATAAACAAGCAACCCAAGTGTCAATTAAAAAAGATTTGG
BRCA2	BRCA2-Asp935Asn-sil	CGAACCCATTTTCAAGAACTCTACCATGGTTTTATATGGAGATACAGGTGACAAACAAGCAACCCAAGTGTCAATTAAAAAAGATTTGG
BRCA2	BRCA2-Lys1180Arg-mut	GCTGATCTTCATGTCATAATGAATGCTCCATCGATTGGTCAGGTAGACAGCAGCAGGCAATTTGAAGGTACAGTTGAAATTAAACGG
BRCA2	BRCA2-Lys1180Arg-sil	GCTGATCTTCATGTCATAATGAATGCTCCATCGATTGGTCAGGTAGACAGCAGCAAACAATTTGAAGGTACAGTTGAAATTAAACGG
BRCA2	BRCA2-Ser1882X-mut	CAGTAAAGTAATTAAGGAAAACAACGAGAATAAATAAAAAATTTGTCAAACGAAAATTATGGCAGGTTGTTACGAGGCATTGG
BRCA2	BRCA2-Ser1882X-sil	CAGTAAAGTAATTAAGGAAAACAACGAGAATAAATCCAAAATTTGTCAAACGAAAATTATGGCAGGTTGTTACGAGGCATTGG
BRCA2	BRCA2-Val2728Ile-mut	GATACCCAAAAAGTGGCCATTATTGAACTTACAGATGGGTGGTATGCTATTAAAGCCCAGTTAGATCCTCCCCTCTTAGCTGTC
BRCA2	BRCA2-Val2728Ile-sil	GATACCCAAAAAGTGGCCATTATTGAACTTACAGATGGGTGGTATGCTGTAAAAGCCCAGTTAGATCCTCCCCTCTTAGCTGTC
BRCA2	BRCA2-Gln2829Arg-mut	GGAAATGTTGGTTGTGTTGATGTAATTATTCAAAGAGCATACCCTATCCGGGTATGATGTATTCTTGAAACTTACCATATATTTC

BRCA2	BRCA2-Gln2829Arg-sil	GGAAATGTTGGTTGTGTTGATGTAATTATTCAAAGAGCATATCCTATCCAGGTATGATGTATTCTTGAAACTTACCATATATTTC
POLE	POLE-Ala31Ser-mut	CCCTTCTTTCACTCAGGGATGATGGCGCCACTTCCTCAGTTTCGTCACTCAAGCGCCTCGAACGGAGTCAGTGGACGGATAAGATGG
POLE	POLE-Ala31Ser-sil	CCCTTCTTTCACTCAGGGATGATGGCGCCACTTCCTCAGTTTCGGCCCTCAAGCGCCTCGAACGGAGTCAGTGGACGGATAAGATGG
POLE	POLE-Pro286Ser-mut	GACATTGAGACGACCAAACTGCCCCTCAAGTTTTCTGATGCTGAGACAGATCAGATTATGATGATTTCCTACATGATCGATGGCCAGG
POLE	POLE-Pro286Ser-sil	GACATTGAGACGACCAAACTGCCCCTCAAGTTTCCTGATGCCGAGACAGATCAGATTATGATGATTTCCTACATGATCGATGGCCAGG
POLE	POLE-Asp301Gly-mut	GCTGAGACAGACCAGATTATGATGATTTCGTACATGATCGGTGGCCAGGTGAGCAGGTGGCTTCTGGGAAGTAAGCTCCTGGG
POLE	POLE-Asp301Gly-sil	GCTGAGACAGACCAGATTATGATGATTTCGTACATGATTGATGGCCAGGTGAGCAGGTGGCTTCTGGGAAGTAAGCTCCTGGG
POLE	POLE-Asn363Asp-mut	GGTTTGAACACGTCCAGGAGACCAAACCCACCATCATGGTCACCTACGACGGAGACTTTTTTGACTG GTGAGTCTGTGTCTTC
POLE	POLE-Asn363Asp-sil	GGTTTGAACACGTCCAGGAGACCAAACCCACCATCATGGTCACCTACAATGGAGACTTTTTTGACTG GTGAGTCTGTGTCTTC
POLE	POLE-Leu424Val-mut	GGTGGGTGAAGAGGGACAGTTACCTTCCTGTGGGCAGTCATAATGTCAAGGCCGCCGCCAAGGCCAAGCTAGGCTATGATCCCG
POLE	POLE-Leu424Val-sil	GGTGGGTGAAGAGGGACAGTTACCTTCCTGTGGGCAGTCATAATCTGAAGGCCGCCGCCAAGGCCAAGCTAGGCTATGATCCCG
POLE	POLE-Ala456Pro-mut	GATGGCCCTGCTCTCTGGCGTTCTCTTCTCAGACTCTGCCCACGTATTCTGTGTCAGATGCTGTCGCCACTTACTACCTGTAC
POLE	POLE-Ala456Pro-sil	GATGGCCCTGCTCTCTGGCGTTCTCTTCTCAGACTCTGGCCACATATTCTGTGTCAGATGCTGTCGCCACTTACTACCTGTAC
POLE	POLE-Phe695Ile-mut	CAGCCAGTCGCAGCGAATACCATCGGATCCAGCACCAGCTCGAGTCAGAGAAGATCCCCCCTTGTTCCCAGAGGGGCCAGCTCGG
POLE	POLE-Phe695Ile-sil	CAGCCAGTCGCAGCGAATACCATCGGATCCAGCACCAGCTCGAGTCAGAGAAGTTTCCCCCCTTGTTCCCAGAGGGGCCAGCTCGG
POLE	POLE-Arg1826Trp-mut	CCCAGTACCACAACATCTATGCAGACAATCAGGTGATGCACTTCTACCGCTGGCTTTGGTCGCCATCCTCTCTGCTTCATGACCC
POLE	POLE-Arg1826Trp-sil	CCCAGTACCACAACATCTATGCAGACAATCAGGTGATGCACTTCTACCGCTGGCTTCGTTCGCCATCCTCTCTGCTTCATGACCC
**Genes**	**Names**	**Primers sequences**
LIG4	LIG4-Fwd	CTGGAGAACAGAATTGCAGA
LIG4	LIG4-Rev	TAGCAATCATATTCACGGGC
XRCC4	XRCC4-Fwd	GAGGCCAGTACAGAAAACAT
XRCC4	XRCC4-Rev	TGGAAAAGTATCCCTGAGGA

Table S2: Odds of Pathogenicity (OddsPath) estimated by performance of classified variant controls. In this assay, five benign and five pathogenic variants of *BRCA1* and *BRCA2* were used as controls allowing the estimation of the evidence strength of our classification according to Brnich *et al*[30] guidelines.

						Note: this estimate "adds 1.0" to the numerator and denominator in order to do a calculation			
# Classified			Proportion pathogenic (Prior, P_1)	Assay result		"+1 proportions" (Posterior, P_2)		OddsPath=$[P_2 \times (1- P_1)] / [(1- P_2) \times P_1]$	
Total	Pathogenic	Benign		Functionally abnormal	Functionally normal	Pathogenic	Benign	Pathogenic	Benign
**10**	**5**	**5**	**0.50**	**5**	**5**	**0.83**	**0.17**	**5.0**	**0.200**

	Odds of Pathogenicity (OddsPath)	Evidence strength equivalent
	<0.0029	BS3_very_strong^
	<0.053	BS3
**Current assay →**	<0.23	BS3_moderate*
	<0.48	BS3_supporting
	0.48-2.1	Indeterminate
	>2.1	PS3_supporting
**Current assay →**	>4.3	PS3_moderate
	>18.7	PS3
	>350	PS3_very_strong

^There are no very strong benign evidence codes included in the Richards, et al. guidance. This category is included here for modeling purposes only.

*Since there are no moderate strength benign evidence codes included in the Richards, et al. guidance, moderate level evidence is equivalent to two instances of supporting-level benign evidence

Table S3: Variants used as silent controls or references with their classification according to different databases.

Benign mutations are shown in green (+), unreported mutations are shown in yellow (?), mutations with conflicting interpretation are shown in orange (+/-), pathogenic mutations are shown in red (-) and intermediate mutations are shown in blue (I).

Gene	Nucleotide change	Amino acid change	Exon	Silent control	Silent reference	UMD database	Clinvar database	BRCA exchange	Characterized variant
BRCA1	c.93C>T	Ile31=	3			?	?	?	Ile31Asn
BRCA1	c.96G>A	Lys32=	3			?	?	?	Ile31Asn
BRCA1	c.442-12C>T		3			?	?	?	Glu149Ala, Thr150Ala
BRCA1	c.450C>G	Thr150=	3			?	?	?	Glu149Ala, Thr150Ala
BRCA1	c.537C>T	Tyr179=	8			?	?	?	Tyr179Cys
BRCA1	c.546G>A	Leu182=	8			?	?	?	Tyr179Cys
BRCA1	c.573T>A	Val191=	8			?	?	?	Val191Asp
BRCA1	c.579G>A	Lys193=	8			?	+	+	Val191Asp, Cys197Cys
BRCA1	c.591C>T	Tyr196=	8			+	+	+	Cys197Cys
BRCA1	c.633A>T	Gly211=	10			?	?	?	Gln210Gln
BRCA1	c.636C>G	Thr212=	10			?	?	?	Gln210Gln
BRCA1	c.840C>A	Ala280=	11			?	?	?	Leu291X
BRCA1	c.873A>G	Leu291=	11			?	?	?	Leu291X
BRCA1	c.1254G>A	Glu418=	11			?	+	+	Tyr422X
BRCA1	c.1266T>C	Tyr422=	11			?	?	?	Tyr422X
BRCA1	c.1386G>A	Gly462=	11			?	+	+	Gly462Arg
BRCA1	c.1404G>A	Lys468=	11			?	+	?	Gly462Arg
BRCA1	c.2928C>T	Asn976=	11			?	?	?	Arg979Cys
BRCA1	c.2937T>C	Arg979=	11			?	?	?	Arg979Cys
BRCA1	c.3243T>C	Asn1081=	11			?	+	+	Leu1080=
BRCA1	c.3261G>A	Gly1087=	11			?	?	?	Leu1080=
BRCA1	c.3594C>G	Leu1198=	11			?	?	?	Gly1201Ser
BRCA1	c.3603T>A	Gly1201=	11			?	?	?	Gly1201Ser
BRCA1	c.3738C>T	Thr1246=	11			?	+	+	Glu1250Lys
BRCA1	c.3750G>A	Glu1250=	11			?	+	+	Glu1250Lys
BRCA1	c.3825A>C	Ile1275=	11			?	?	?	Ile1275Val
BRCA1	c.3834G>A	Lys1278=	11			?	+	+	Ile1275Val
BRCA1	c.4173G>A	Ile1391=	12			?	?	?	Thr1394Ile
BRCA1	c.4179C>A	Thr1393=	12			?	?	?	Thr1394Ile
BRCA1	c.4503C>T	Cys1501=	15			+/-	+	+	Asp1506Asn, Asp1506Glu
BRCA1	c.4518T>C	Asp1506=	15			?	+	+	Asp1506Asn, Asp1506Glu
BRCA1	c.4752C>T	Ala1584=	15			?	+	+	Glu1586X

BRCA1	c.4758G>A	Glu1586=	15			?	?	?	Glu1586X
BRCA1	c.4791C>G	Thr1597=	16			?	?	?	Gln1604X, Gln1604=
BRCA1	c.4809C>A	Pro1603=	16			?	?	?	Gln1604=
BRCA1	c.4812A>G	Gln1604=	16			+	+	+	Gln1604X
BRCA1	c.5194-28C>G		20			?	?	?	c.5194-2A>G
BRCA1	c.5194-10T>G		20			?	?	?	c.5194-2A>G
BRCA1	c.5256A>T	Ala1752=	20			?	?	?	Ala1752Thr, Ala1752Pro
BRCA1	c.5268G>A	Gln1756=	20			?	+	+	Ala1752Thr, Ala1752Pro
BRCA1	c.5310G>T	Gly1770=	21			?	+	+	Gly1770Val
BRCA1	c.5313C>G	Pro1771=	21			?	?	?	Gly1770Val
BRCA1	c.5415C>T	His1805=	23			?	+	?	Pro1812Ala
BRCA1	c.5436A>G	Pro1812=	23			+/-	?	?	Pro1812Ala
BRCA2	c.654A>G	Glu218=	8			?	?	?	Glu218Lys
BRCA2	c.669T>C	His223=	8			?	?	?	Glu218Lys
BRCA2	c.2139G>A	Gln713=	11			?	?	?	Asn719Lys
BRCA2	c.2157T>C	Asn719=	11			?	?	?	Asn719Lys
BRCA2	c.2796C>T	Asp932=	11			?	?	?	Asp935Asn
BRCA2	c.2805T>C	Asp935=	11			?	+	+	Asp935Asn
BRCA2	c.3510C>T	Ala1170=	11			+/-	?	?	Lys1180Arg
BRCA2	c.3540G>A	Lys1180=	11			?	?	?	Lys1180Arg
BRCA2	c.5646A>C	Ser1882=	11			?	?	?	Ser1882X
BRCA2	c.5655C>T	Cys1885=	11			?	?	?	Ser1882X
BRCA2	c.8184T>A	Val2728=	18			?	?	?	Val2728Ile
BRCA2	c.8187G>A	Lys2729=	18			?	+	+	Val2728Ile
BRCA2	c.8478C>T	Tyr2826=	19			?	?	?	Gln2829Arg
BRCA2	c.8484A>C	Ile2828=	19			?	?	?	Gln2829Arg
**Gene**	**Nucleotide change**	**Amino acid change**	**Exon**	**Silent control**	**Silent reference**	**Exac**	**Clinvar database**	**OncoKb**	**Characterized variant**
POLE	c.93A>C	Ala31=	2			+	+	?	Ala31Ser
POLE	c.105G>C	Leu35=	2			+	?	?	Ala31Ser
POLE	c.864T>C	Ala288=	9			?	+	?	Pro286Ser
POLE	c.874C>T	Asp291=	9			?	?	?	Pro286Ser
POLE	c.891C>G	Ser297=	9			?	?	?	Asp301Gly
POLE	c.900C>T	Ile300=	9			?	+	?	Asp301Gly
POLE	c.1089C>T	Asn363=	11			+	+	?	Asn363Arg
POLE	c.1092G>A	Gly364=	11			+	+	?	Asn363Arg
POLE	c.1272C>G	Leu424=	13			?	+	?	Leu424Val
POLE	c.1278G>C	Ala426=	13			?	+	?	Leu424Val
POLE	c.1360-6C>T		14			+	+	?	Ala456Pro

POLE	c.1371G>A	Thr457=	14			+	+	?	Ala456Pro
POLE	c.2070G>C	Leu690=	19			?	?	?	Phe695Ile
POLE	c.2085C>T	Phe695=	19			?	+/-	?	Phe695Ile
POLE	c.5448C>T	Asn1815=	40			?	?	?	Arg1826Trp
POLE	c.5478G>T	Arg1826=	40			+	+	?	Arg1826Trp
